# Editorial on Special Issue: “Dynamics of Gels and Its Applications”

**DOI:** 10.3390/gels8120805

**Published:** 2022-12-08

**Authors:** Yiming Yang, Di Jia

**Affiliations:** 1 Beijing National Laboratory for Molecular Sciences, State Key Laboratory of Polymer Physics and Chemistry, Institute of Chemistry, Chinese Academy of Sciences, Beijing 100190, China; 2 The University of Chinese Academy of Sciences, Beijing 100049, China; 3 Key Laboratory of Science and Technology on High-Tech Polymer Materials, Institute of Chemistry, Chinese Academy of Sciences, Beijing 100190, China

Gels are polymer networks swollen in various solvents. They are not only abundant in nature in the form of hydrated tissues, but also on your lunch plate in the form of Tofu or noodles. Despite strong efforts and significant advances, the dynamical behavior of hydrogels is yet to be fully understood. In particular, the relationship between the microscopic dynamics at the monomeric level and the macroscopic elastic properties at the level of the bulk gels is yet to be fully established, and their applications still need to be explored further. Fortunately, in this study, we obtained 11 papers. The Editorial Team thanks all the contributors.

The papers in this Special Issue effectively represent the diverse features of the dynamics of gels from fundamental studies to applications. To obtain a comprehensive understanding of the dynamical properties of the gels, various tools are necessary for characterization purposes at different length scales. Dhakal et al. studied the effects of the deformation on stretched gel dynamics parallel and perpendicular to the stretching direction using dynamic small-angle light scattering (DSALS) setup [1]. Their work showed that DSALS is an effective tool used to evaluate local hydrogel dynamical responses to deformation. Zhou et al. characterized the ionization of poly(ethylene glycol), which is one of the most famous hydrophilic polymers used in hydrogels. They revealed that poly(ethylene glycol) chains exhibited polyelectrolyte-like expansion behavior and shrinkage under different salt concentrations, which enhances the possibility of poly(ethylene glycol)-based gels being utilized in more complicated environments [2]. Polyelectrolyte gels exhibited intricate dynamical behavior compared to the uncharged gels. Jia et al. summarized the relationship between microscopic properties (such as the gel diffusion coefficient) and macroscopic properties (such as elasticity and the friction coefficient for polyelectrolyte gels). In addition, the electrostatic coupling between charged moieties and their ion clouds (which significantly modifies the elastic diffusion coefficient of gels) as well as various scaling laws were also discussed in depth [3].

The dynamic properties of gels can further fulfill the requirements for biomedical applications. Dumitrascu et al. showed that alginate hydrogels crosslinked with nickel (II) or cobalt (II) can mimic IMAC preferential protein binding and can fully degrade to release the payload in vivo [4]. Samimi Gharaie et al. designed a new bio-ink formula consisting of laponite, graphene oxide, and alginate. The double physical-crosslinked gels showed stretchable, soft, but durable properties, as well as tunable electro-conductivity after extrusion printing and crosslinking [5]. Other than robust gels for biomedical applications, low viscous gels were essential to ophthalmic diseases. The design of these gels and their mechanical properties were discussed in detail [6,7]. Meanwhile, Huang et al. controlled the mechanical properties of poly(methyl methacrylate) hydrogels via dynamical physical crosslinks from pillar[5]arene-based host–guest interactions [8]. This host–gust complex can dissociate to dissipate energy upon loading and reform when loading is removed. Moreover, the dynamic control of gel formation creates hierarchical structures that are difficult to obtain in covalent gel systems. The control of the sol–gel process significantly influenced the microstructure of gels. Dong et al. showed composite gel from textile waste fiber and poly(vinyl alcohol), crosslinked first by the formation of hydrogen bonding on the surface of fiber followed by the covalent linkage of the acetal functional group by glutaraldehyde. The merit properties of such aerogels from textile waste fiber expands the application perspectives associated with recycled materials [9]. Racles et al. demonstrated a new method to stabilize the emulsion aqueous phase within toluene sol, followed by the UV-triggered thiolene crosslinking of vinyl-polysiloxane to form emulsion gels [10]. Moreover, Guastaferro et al. showed fully physical crosslinked aerogel from agarose through the sol–gel process followed by supercritical drying or freeze-drying [11]. High-polymer-content gel showed the least shrinkage and largest surface area for both drying methods. Moreover, Tarasova et al. showed a unique gelation method, which helped to realize the sequences of two or more stages in one reaction [12].

We hope that this Special Issue will promote a better understanding of the dynamics of gels and its applications. We thank all the scientists who collaborated with our plan of the “Dynamics of Gels and Its Applications” Special Issue.
